# Oral Supplementation of n-3 Polyunsaturated Fatty Acids (n-3-PUFA) Can Prevent TBI-Induced Visual, Motor, and Emotional Deficits in Mice

**DOI:** 10.1007/s12035-025-05019-9

**Published:** 2025-05-09

**Authors:** Koushik Mondal, Ashlyn A. Gary, Anisha Dash, Nobel A. Del Mar, Daniel J. Stephenson, Charles E. Chalfant, Anton Reiner, Barry Sears, Nawajes Mandal

**Affiliations:** 1 Department of Ophthalmology, The University of Health Science Centre, 930 Madison Ave., Suite 718, Memphis, TN 38163 USA; 2 Miller School of Medicine, University of Miami, Miami, FL USA; 3 ETSU Quillen College of Medicine, Mountain Home, Johnson City, TN 37684 USA; 4 Departments of Medicine and Cell Biology, University of Virginia School of Medicine, Charlottesville, VA 22903 USA; 5 Research Service, Richmond VA Medical Center, Richmond, VA 23298 USA; 6 Department of Anatomy and Neurobiology, The University of Health Science Centre, Memphis, TN 38163 USA; 7 Inflammation Research Foundation, Peabody, MA 01960 USA; 8 Department of Pharmaceutical Sciences, College of Pharmacy, The University of Health Science Centre, Memphis, TN 38163 USA; 9 Memphis VA Medical Center, Memphis, TN 38104 USA; 10 Molecular Diagnostics Laboratory, Department of Basic & Translational Research, Saroj Gupta Cancer Centre & Research Institute, Kolkata, WB 700 063 India

**Keywords:** Traumatic brain injury (TBI), N-3 PUFA, Ceramide, Visual deficits, Emotional deficits, Neuroinflammation

## Abstract

Traumatic brain injury (TBI) causes neuroinflammation and can generate long-term pathological consequences, including motor and visual impairments, cognitive deficits, and depression. In our previous study, we found that *Fat1*^+^-transgenic mice with higher endogenous n-3 polyunsaturated fatty acids (n-3 PUFA) were protected from post-TBI behavioral deficits and exhibited reduced levels of TBI-induced microglial activation, inflammatory factors, and sphingolipid ceramide, a lipid mediator of inflammation and cell death. This study’s objective was to evaluate if feeding n-3 PUFA (EPA and docosahexaenoic acid, DHA 2:1) could restrict the elevation of ceramide in brain tissue and prevent TBI-mediated sensory-motor and behavioral deficits. Wildtype C57/BL6 mice were gavage pre-fed with PUFA (EPA: DHA = 2:1) at 500 mg/kg body weight/week for 2 weeks before and 4 weeks after exposure to left side focal cranial air-blast (50 psi) TBI or sham-blast (0-psi). Saline-gavaged mice served as controls. Following blast injury, various motor, visual, and behavioral tests were conducted, and brain tissues were collected for histological and biochemical assays. Lipidomics analysis confirmed a significant elevation of EPA in the plasma and brain tissue of PUFA-fed mice. TBI-Blast brain tissues were found to have elevated ceramide levels in control mice but not in PUFA-fed mice. Moreover, PUFA-fed mice demonstrated protection against motor impairment, photoreceptor dysfunction, depression, oculomotor nerve degeneration, and microglia activation in the optic tract. Our results demonstrate that EPA-mediated suppression of ceramide biosynthesis and neuroinflammatory factors in PUFA-fed mice is associated with significant protection against the visual, motor, and emotional deficits caused by TBI.

## Introduction

Traumatic brain injury (TBI), caused by a violent jolt or blow to the head, is a significant risk factor for neurodegenerative diseases such as Alzheimer’s and Parkinson’s Disease [1]. Common reasons for brain injury involve forced contact between the players in sports, exposure to blast injuries in warfare, and motor vehicle crashes [1, 2]. Based on the Glasgow Coma Scale Score (GCS), the severity of TBI has been classified as mild, moderate, and severe [3]. While mild traumatic brain injury (mTBI) was once thought to be benign compared to severe TBIs, accumulating evidence suggests its lasting neurological impacts, including deficits in memory, cognition, sleep, vision, and hearing [4–6]. The injury associated with TBI is divided into primary injury and secondary injury. The mechanical damage associated with the immediate insult is primary injury. In contrast, the onset of physiological changes and biochemical cascades of events leading to neuronal cell death and functional impairments are called secondary injury [7, 8]. These pathophysiological changes progressively develop after the primary injury, impacting the brain’s sensory, cognitive, motor, and emotional functions [9, 10]. The heterogeneous nature of mTBI pathophysiology, including the release of inflammatory cytokines, neurotransmitters, and biochemical mediators, further complicates its understanding and therapeutic management. Primary injury is refractory to therapeutic strategies; thus, preventing the development of secondary injuries is considered the most effective approach to managing mTBI.

Understanding various molecular and cellular factors influencing secondary injury is of the utmost necessity for the prognosis and identification of therapeutic targets in mTBI. Although several pharmacological agents are being used in TBI’s acute and post-acute phases, better management is necessary due to its heterogeneous pathophysiology [11]. Our research aims to determine molecular mechanisms and factors that align closely with the disease’s pathophysiology with standardized TBI preclinical models that play protective roles in developing potential future therapies. In a previous study, we used a transgenic mouse model system to investigate probable biochemical markers in mTBI [12]. We chose the closed-head injury model for its ability to closely replicate the injuries seen in patients compared to other preclinical models of mTBI [13]. In this closed-head injury model, we observed that the pathophysiology of mTBI was associated with the secretion of inflammatory cytokines and increased levels of bio-mediators, including inflammatory sphingolipids (SPL) such as ceramides in brain tissues [12]. These pathological changes were associated with the damage of the oculomotor nucleus and concurrent microglial activation at the optic tract [12]. Emerging research suggests that perturbations of SPL components are involved in neuroinflammatory and neurodegenerative disease processes [14–18].

N-3 polyunsaturated fatty acid (n-3 PUFA) containing lipids are known to be anti-inflammatory [19, 20]. Our prior investigation revealed that *Fat1*^+^-transgenic mice containing elevated levels of systemic n-3 PUFA, effectively restricted upregulation of pan-inflammatory agents, including inflammatory cytokines and ceramides after blast injury [12]. Moreover, these mice exhibited preserved oculomotor nuclei, reduced microglial activation, and increased resistance to decline in visual acuity, motor function, and depression compared to wildtype mice [12]. Several preclinical studies have suggested that n-3 PUFA [eicosapentaenoic acid, EPA (20:5n3); docosahexaenoic acid, DHA (22:6n3)] could be a promising therapeutic approach to mitigating neuroinflammation and neurodegeneration following TBI [21–24]. EPA and DHA can play a vital role in dampening secondary injury by diminishing TBI-mediated excitotoxicity, microglial activation, axonal damage, and apoptosis [25]. With the demonstrated efficacy of n-3 PUFA in different preclinical studies, human clinical studies have also started testing their effectiveness [26, 27]. Our prior study indicated that increased levels of n-3 PUFA in transgenic mice with the *Fat1*^+^ gene, which codes for n-3 fatty acid desaturase, conferred neuroprotection potentially via regulation of SPL. This regulation led to a reduction in ceramide toxicity and neuroinflammation [12]. To further confirm n-3 PUFA’s effectiveness in limiting pathological changes in SPL and its translational value, the present study aims to investigate the oral supplementation of n-3 PUFA in preventing visual, motor, and emotional deficits following mTBI.

## Materials and Methods

### Animals and Feeding with n3-PUFA

All procedures were performed according to the Association for Research in Vision and Ophthalmology Statement for the Use of Animals in Ophthalmic and Vision Research, the National Institutes of Health, the Society for Neuroscience, and the University of Tennessee Health Science Center Guidelines for Animals in Research. All procedures, tissue harvests, and euthanasia methods were reviewed and approved by the UTHSC Institutional Animal Care and Use Committee (UTHSC IACUC). C57BL/6 J mice were born and raised in the UTHSC LACU (Laboratory Animal Care Unit) vivarium and maintained from birth under cyclic light (50–100 lx, 12 h on/off, 7 a.m. to 7 p.m. CST).

**Fig. 1:**
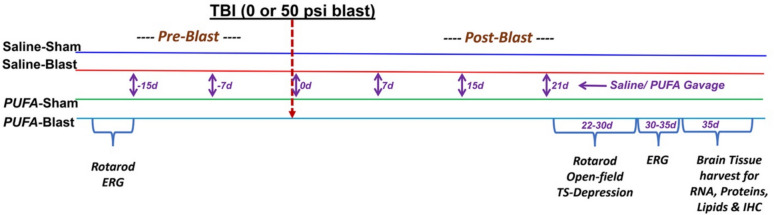
Schematic diagram of the experimental timeline, study groups, treatments, observations and tests, and tissue harvest

Since mammals cannot endogenously synthesize n-3 PUFA from the more abundant n-6 PUFA found in typical human diets, this study administrated a n-3 PUFA dietary intervention. An equal number of mice were pre-fed mice with n-3 PUFA at a dose of 500 mg/kg body weight/week or an equal volume of saline by gavage (one gavage per week) for 2 weeks. The source of n-3 PUFA was Dr. Sears’ Omega Rx 2 Fish Oil Liquid from Zone Labs Inc., Peabody, MA 01960. After two weeks, mice were exposed to blast or TBI and feeding continued for 4 more weeks after the blast. Saline-gavage-fed mice served as controls. Mice were divided into 4 groups (6–8 mice per group) and exposed to mild TBI with a 50-psi blast (Blast) or 0-psi blast (Sham) on the left side of the skull between the ear and eye. Following blast injury, mice were grouped as Saline-Sham, Saline-Blast, PUFA-Sham, and PUFA-Blast. Mice were assessed for motor function, visual function, cognitive function, and depression at various time points before (Pre-Blast) and after the blast (Post-blast) (Fig. 1).

Experimental mice were divided into 4 groups and exposed to mild TBI with a 50-psi blast (Blast) or 0-psi blast (Sham) on the left side of the skull between the ear and eye. C57/BL6 mice were grouped as saline-fed (normal chow with saline gavage) and PUFA-fed (normal chow and PUFA gavage), before the 15 days of blast injury. After blast injury, mice were grouped further as Saline-Sham, Saline-Blast, PUFA-Sham, and PUFA-Blast. Mice were tested for motor, visual, cognitive function, and depression at various time points before (Pre-Blast) and after the blast (Post-blast). In addition, brain tissues were surgically extracted after 35 days of blast injury for biochemical and molecular biology experiments. OKN, Optokinetic nystagmus reflex; ERG, Electroretinography. IHC, Immunohistochemistry.

### TBI Methods

As described before, mice in this study were exposed to mild TBI caused by an air blast [10, 28]. The air blast was generated by a mounted air canon system attached to a modified paintball gun (Invert Mini, Empire Paintball, Sewell, NJ). A 0-psi blast served as the control and was termed as a “sham blast.” In all of the experiments, this control was compared to 50-psi blasts. Mice were divided into four groups: Saline-Sham, Saline-Blast, PUFA-Sham, and PUFA-Blast. Mice were given anesthesia, Avertin (400 mg/kg of body weight), and then subjected to the air-pressure blast between their eye and ear on the left side of the cranium. Before the blast, the targeted region of the head was shaved and marked with a white dot painted in the middle. A Plexiglass tube with a 7.5 mm diameter hole that faced the barrel of the blast cannon was used to position the parietal region of the mouse accurately. A foam rubber sleeve secured and stabilized the animal inside the tube. A transducer set the specific air pressure for the blast, which was analyzed using a Labview software system (National Instruments, Austin, TX).

### Electroretinogram

Using the Diagnosys LLC Celeris electroretinogram (ERG) system, recordings were gathered from the scotopic threshold ERG (Diagnosys LLC, Lowell, MA). To prepare the mice, they were exposed to total darkness overnight and then recorded under dim red light for the ERG recording. Mice were injected intraperitoneally with ketamine (100 mg/kg body weight) and xylazine (5 mg/kg body weight) for anesthesia. To dilate the pupil, mice were given one drop of 10% (v/v) phenylephrine onto the cornea and then another drop of 0.5% (v/v) proparacaine HCl for local anesthesia. During the recording, a heating pad at 37⁰C kept the mice warm. For ERG measurement, designated electrodes were placed on each cornea, and the ERG was performed using the TOUCH/TOUCH protocol developed by the manufacturer. Scotopic ERG responses were recorded in the dark and in dark-adapted mice with four flash stimuli at intensities of 0.01, 0.1, 1, and 10 cd.s/m2. The a-wave amplitude was measured from the pre-stimulus baseline to the a-wave trough, while the b-wave amplitude was measured from the trough of the a-wave to the peak of the b-wave. The photopic ERG was recorded after mice underwent light adaptation (3 min at 10 cd.s/m2), followed by two flash stimuli at intensities of 3 and 10 cd.s/m2, along with a 10 Hz flicker response. The a- and b-wave amplitudes were measured pre-blast and one month after the blast.

### Open-Field Behavior Assessment

A Noldus EthoVision video tracking system was used to record mouse movements during an automated 30-min open-field behavior assessment (Noldus Information Technology, The Netherlands). This behavioral test evaluates anxiety and locomotion in mice by analyzing their movement patterns in an open arena, measuring variables such as center rest proportion (time spent in the middle, indicating anxiety levels), stops per distance (pausing frequency, reflecting movement hesitation), and path curvature (walking trajectory, assessing motor control). Another software, the SEE software of Drai and Golani, helped analyze the mice’s motor behavior from the recordings [29]. As determined from prior published protocols, open field sessions were held 22 to 30 days post-blast injury [30].

#### Rotarod-Motor Test

The Rotarod test is used to evaluate motor coordination and balance. The rotarod consists of a circular rod turning at a constant or increasing speed. Animals were placed on the rotating rod to try to remain on it rather than fall onto a platform some 30 cm below. The rod initially rotates at a constant speed of 4 rpm for positioning the animals in their respective lanes, and the speed was increased to 20 rpm over 4 min [31]. This speed was maintained for another 2 min. Each testing session consisted of 3 runs, at least 10 min apart. Latency to fall and speed of the rotarod at the time of fall were recorded. Values for the 3 runs were used to obtain an average for each mouse.

#### Tail Suspension Depression Test

As previously described, a tail suspension depression test was conducted to assess depression-like behavior in mice [10, 28]. Each mouse was positioned facing the ground with its tail attached to a solid surface and the rest of its body suspended in the air. The test lasted for 5 min, and a video tracking system collected responses from a computer. Then, an automated software (FreezeFrame, Coulbourn, Whitehall, PA, USA) quantified this data. Immobility dictated a depressive-like state and was used to measure the behavioral score. A higher score means a longer period of immobility over the testing period. These data were graphed as collective immobility per minute over the 5-min test.

### Immunohistochemical Analysis

Immunohistochemical analyses on fixed brain tissues about 30 days post-TBI helped to determine the effects of TBI on the following: 1) the motor neurons of the oculomotor nucleus using the cholinergic neuron marker choline acetyltransferase (ChAT) for immunolabeling; and 2) microglia activity in the optic tract detected through immunostaining for ionized calcium-binding adapter molecule-1 (IBA-1). Mice were anesthetized with Avertin (0.2 mL/g body weight), chests were opened, and the heart was injected with 0.1 mL of heparinized saline (800 USP units/mL). Next, they were transcardially given 40 mL of 0.9% NaCl in 0.1 M sodium phosphate buffer at a pH 7.4 (PB) and then followed with 200 mL of 4% paraformaldehyde, 0.1 M lysine-0.1 M sodium periodate in 0.1 M PB at pH 7.4 (PLP). The animals’ brains were removed and stored for a minimum of 24 h in a 20% sucrose/10% glycerol solution at 4 °C. A pin was inserted longitudinally into the right brain to help distinguish the right and left sides of the brain. The frozen and fixed brain tissues were transversely sectioned on a sliding microtome at 35 µm. Sections from each brain were gathered and stored as 12 separate series in 0.1 M PB with 0.02% sodium azide. Using a goat polyclonal antibody (Chemicon #AB144) and the peroxidase-anti peroxidase method as done in previous studies, we immunolabeled brain tissues for ChAT to see the effects on the oculomotor nucleus [32]. Images of the oculomotor nucleus were captured at a standardized level using Image J. The ChAT + perikarya were counted, and the nucleus area was quantified blinded as to the group. Brain tissue sections at a standardized level of the optic tract were similarly immunolabeled with rabbit anti-IBA1 (Wako Chemicals #019–19741) in order to detect the microglia using the peroxidase-anti peroxidase method [32, 33]. Using a rounded cell body, increased intensity of IBA1 immunolabeling, and retracted processes as morphological factors dictating a transformation to an activated phenotype, the number of microglia per unit area of the optic tract was counted blinded as to the group.

### Analysis of Polyunsaturated Fatty Acids (PUFA)

The analysis of polyunsaturated fatty acids was completed at the Lipidomic facility at the University of Virginia, Charlottesville, VA. Using methods from previous studies, we measured and analyzed PUFA from the brain, liver, and plasma of Saline-Sham, Saline-Blast, PUFA-Sham, and PUFA-Blast mice 35 days post-TBI [34].

### Sphingomyelinase Assay

Using a previously published protocol and the Amplex Red Sphingomyelinase Assay kit (Invitrogen, Carlsbad, CA), acidic and neutral sphingomyelinase (aSMase and nSMase, respectively) activity was measured from extracted proteins of the brain tissues of Saline-Sham, Saline-Blast, PUFA-Sham, and PUFA-Blast mice 35 days post-TBI [35]. Proteins from the frozen homogenized brain tissue samples were extracted in 100 µL of phosphate buffer saline (PBS, pH 7.4) with protease inhibitor cocktail, which have broad specificity to inhibit serine, cysteine, and metalloproteases, and the protein contents were measured by BCA assay (ThermoFisher Scientific) [35]. In this enzymatic assay, the cascade of enzymatic reaction is carried out in the reaction mixture following the manufacturer’s protocol. SMase present in the sample first hydrolyzes the sphingomyelin (supplied in the reaction) to yield ceramide and phosphorylcholine. Next, phosphorylcholine is hydrolyzed with the action of alkaline phosphatase to form choline. Then, choline is oxidized by choline oxidase to betaine and H_2_O_2_. Finally, H_2_O_2_, in the presence of horseradish peroxidase, reacts with Amplex® Red reagent in a 1:1 stoichiometry to generate the highly fluorescent product, resorufin, which is measured at excitation range 530–560 nm and emission detection at 590 nm, respectively, following the manufacturer’s protocol. The nSMase activity was measured at the near-neutral pH optima (pH ~ 7.4). Whereas aSMase activity was measured in two steps where aSMase reaction was performed at a lower pH, (in 50 mM sodium acetate, pH 5.0), and then the pH was raised to 7.0–8.0 (by adding an equal volume of 100 mM Tris–HCl, pH 8.0) to allow detection with the Amplex® Red reagent. The enzymatic activity is represented as a Fluorescence Unit (FU) in the measured protein concentration (µg of protein).

### Analysis of SPL

Following previous studies’ methodology, mouse brain SPL were analyzed 35 days following TBI using established protocols [36–39]. The samples were dissolved in CH_3_OH:CHCl_3_ (2:1). The internal standards of 500 pmol each for SPL metabolites were bought from Avanti Polar Lipids and dissolved in a cocktail of C_2_H_5_OH:CH_3_OH:H_2_O (7:2:1). This cocktail was added to the samples until reaching a total combined volume of 2020 µl. As in our prior published article, the methodology of sample preparation and processing remained identical (43). SPL were sorted out through reverse phase liquid chromatography (LC) utilizing a Supelco 2.1 (i.d.) × 50 mm Ascentis Express C18 column (Sigma, St. Louis, MO) and a solvent system with a flow rate of 0.5 mL/min. The column was first washed out for 30 s with a solvent of 95% Mobile phase A1 (CH_3_OH/H_2_O/HCOOH, 58/41/1, v/v/v, with 5 mM ammonium formate) and 5% Mobile phase B1 (CH_3_OH/HCOOH, 99/1, v/v, with 5 mM ammonium formate). The samples are then eluted in the LC. Following the injection of the sample in the column, the A1/B1 ratio was steady at 95/5 for 2.25 min. Then, a linear gradient to 100% B1 over 1.5 min was held here for 5 min, followed by a rapid 0.5 min gradual return to the original 95/5 A1/B1. Before the subsequent run, the column was equilibrated again for 30 s with 95/5 A1/B1. Various species of ceramides (Cer), sphingomyelin (SM), hexosyl-ceramide (Hex-Cer), and SPL categories such as sphingosine (Sph), S1P, DHS1P, and dihydro-sphingosine (DH-Sph) were determined by their mass-to-charge(m/z) ratio and retention time and quantified as reported in this article [36].

### Statistical Analysis

GraphPad Prism 10 software was used for data analysis. Depending on the experiment, a Student’s *t*-test or a one- or two-way ANOVA was used for statistical analyses with appropriate correction measures. For the tail suspension test, a chi-square was performed. A *P value* < 0.05 was considered statistically significant.

## Results

### Assessment of Major n3 and n6 PUFA Levels in Plasma and Brain

**Fig. 2:**
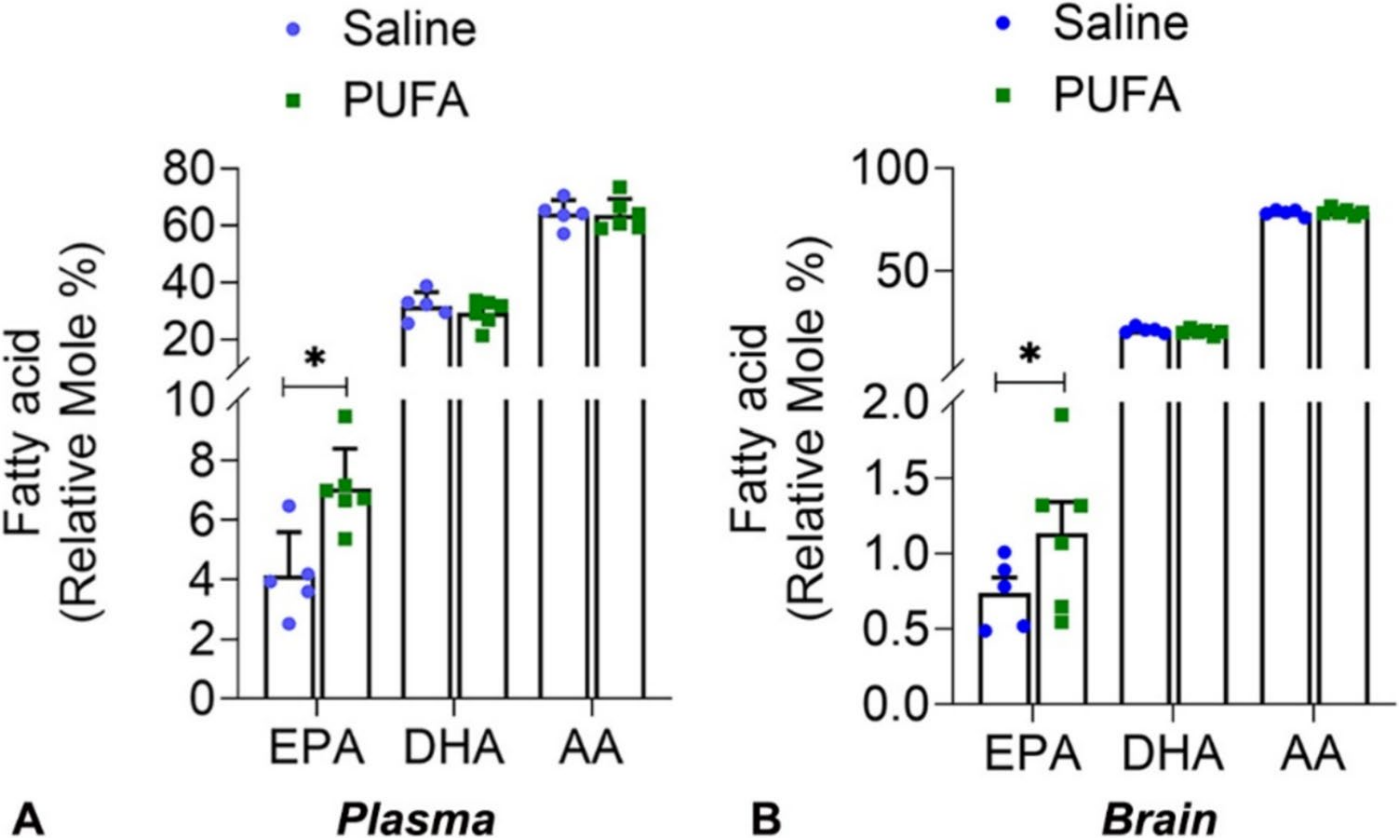
Analysis of different Polyunsaturated fatty acids (PUFAs) in the tissues of saline-fed (Saline) and PUFA-fed (PUFA) mice. Relative mole percentages of PUFA [20:5n3, Eicosapentaenoic acid (EPA); 22:6n3, Docosahexaenoic acid (DHA); 20:4n6 Arachidonic acid (AA)] in the Plasma (**A**) and brain (**B**) of saline and PUFA- fed mice. Data are presented as Mean ± SEM, and levels of significance are shown as *P values* (*n* = 5; *p < *0.05*, *t-test*)

We compared both plasma and brain lipid profiles to compare the systemic effect of oral supplementation on the PUFA levels in PUFA-fed mice relative to saline-fed mice. The PUFA-fed mice were given n-3 PUFA supplementation for 6 weeks of 500 mg/kg/week with a ratio of EPA:DHA = 2:1. Using mass spectrometric lipidomics analysis, we observed a significant increase in the levels of EPA in the plasma and brain tissues in PUFA-fed mice (Fig. 2). The relative mole percentages of EPA were increased in both tissues in PUFA-fed compared to their saline-fed counterparts. There were no significant differences in the levels of major n-3 PUFA, DHA, or the major n-6 PUFA, arachidonic acid (AA), between the plasma or brain of PUFA-fed mice and saline-fed mice (Fig. 2). Our data indicates that a diet supplemented with oral n-3 PUFA causes changes to mice’s brain and plasma lipid profiles, leading to a systemic increase in EPA levels only, and provides a valuable model to study the effects of increased EPA in conditions such as mild TBI.

### Retinal Function Test by Electroretinography

**Fig. 3:**
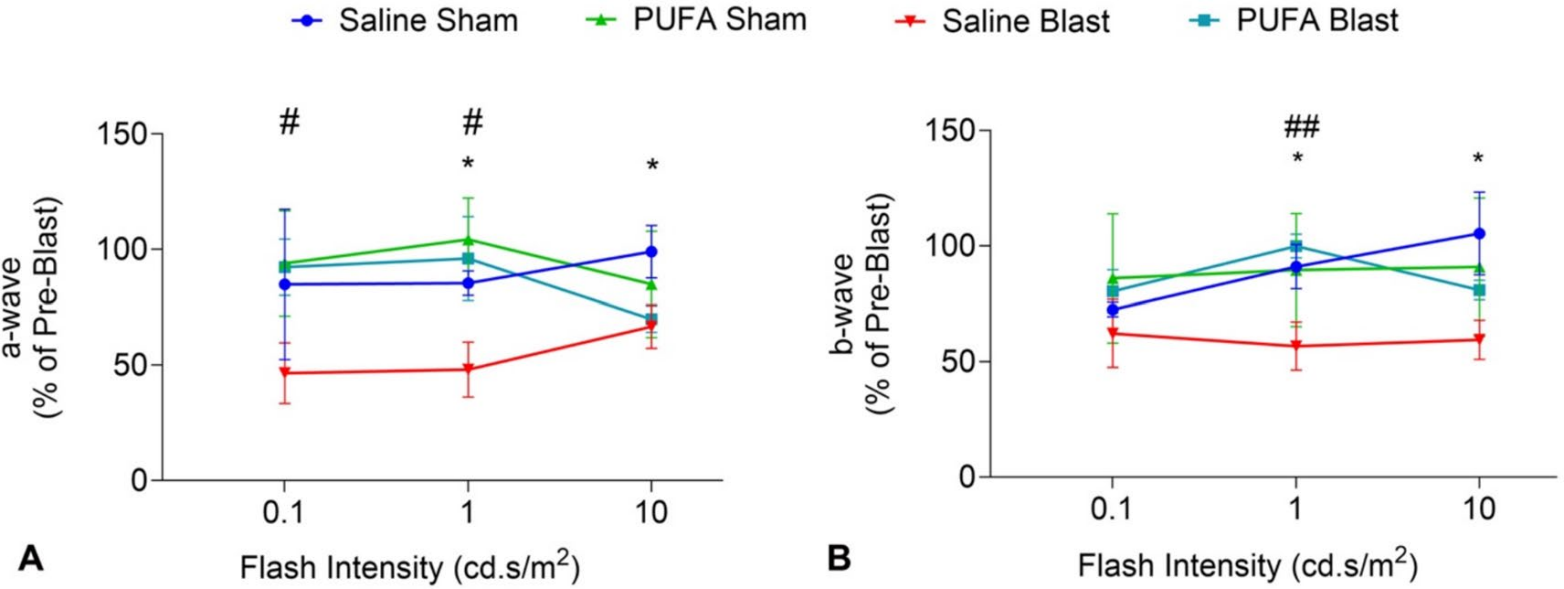
Retinal function assay by Electroretinogram of saline-fed (Saline) and PUFA-fed (PUFA) mice treated with 0-psi (sham) or 50-psi (blast) TBI. Pre-blast a-wave and b-wave amplitude values served as reference for each group. Data are presented as percentages value of pre-blast in a-wave (**A**) and b-wave (**B**) (*n* = 4 Saline-Sham, 5 Saline-Blast, 4 PUFA-Sham, 4 PUFA-Blast (*p < 0.05 (* indicates Saline Sham vs Saline Blast); ^#^*p* < *0.05*, ^##^*p* < *0.01* (.^#,##^ indicates PUFA Blast vs Saline Blast; ANOVA; *t-test*)

We measured retinal function by recording dark-adapted scotopic a-wave and scotopic b-wave from each mouse at 15 days pre-blast and 30 days post-blast. Our findings indicate a significantly reduced level of the scotopic a-wave in Saline-Blast and PUFA-blast mice compared to sham mice (Fig. 3A). Similarly, the scotopic b-wave was significantly decreased in Saline-Blast mice compared to Saline-Sham mice. In contrast, the scotopic b-wave was increased in PUFA-Blast mice when compared to PUFA-Sham mice (Fig. 3B). Of note, we observed a rescue effect at lower light intensities: the difference between sham and blast conditions was substantial in saline-fed mice but either absent or minimal in PUFA-fed mice across all light intensities. Overall, Saline-Blast mice exhibited significantly reduced levels of both a-wave and b-wave amplitudes compared to PUFA-Blast mice. These results suggest that mild TBI adversely affects retinal function, but oral intake of PUFA might offer some level of protection to photoreceptor function.

### Behavioral and Motor Function Assays

#### Open-Field Test Evaluation

**Fig. 4:**
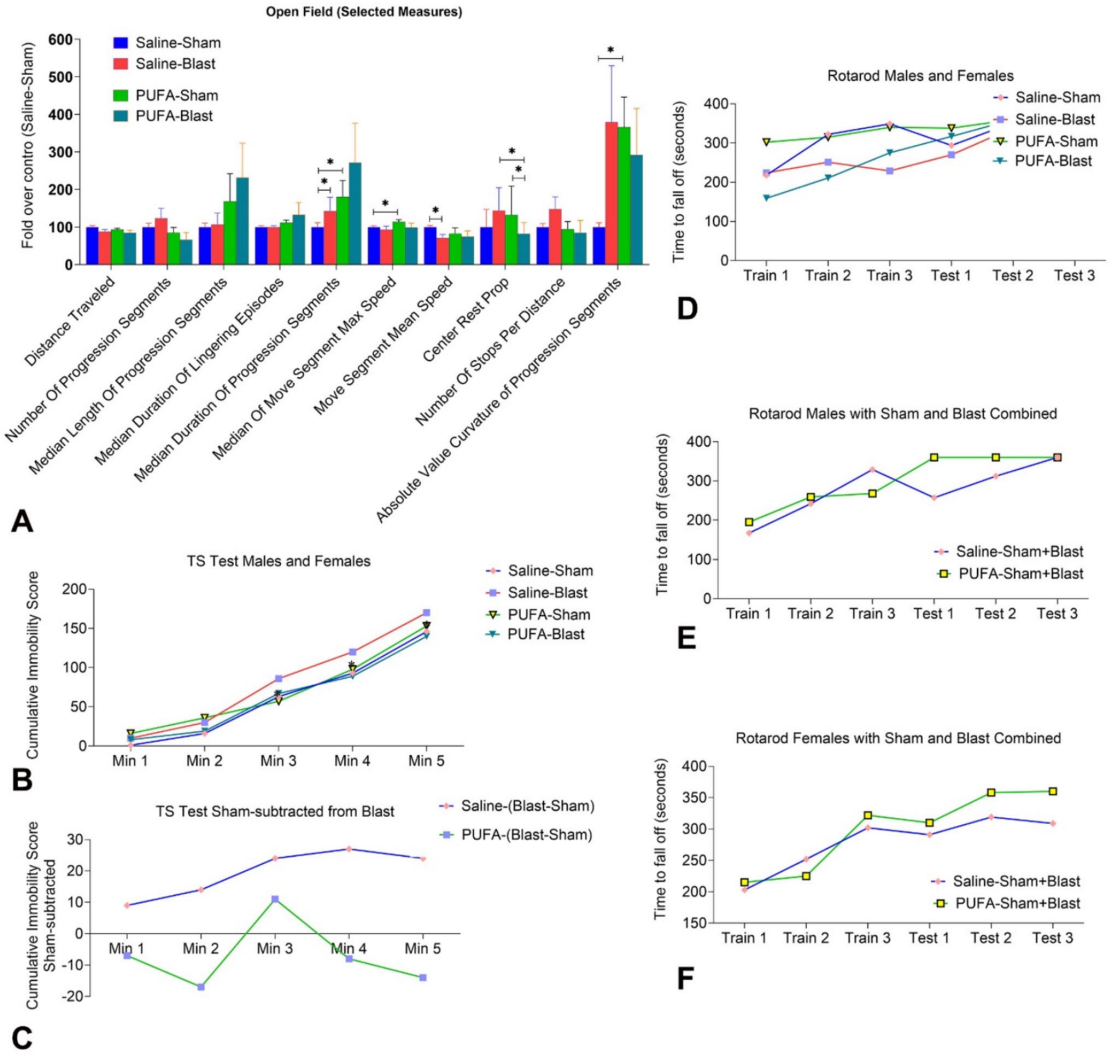
Analysis of open field behavior, motor coordination, and depression in saline-fed (Saline) and PUFA-fed (PUFA) mice with (50-psi, blast) or without (0-psi, sham) mild TBI. Mice were divided into four groups, Saline-Sham, Saline-Blast, PUFA-Sham, and PUFA-Blast, each containing 6–8 mice. Open-field motor testing was conducted, and data are represented as percentages values of fold-over control (Saline-Sham) (**A**). The Tail suspension testing was conducted, and data are presented as line graphs of cumulative immobility per minute over the 5 min test, in which more immobility reflects more depression-like behavior (**B–C**). The Rotarod Motor Test was conducted, and the data are presented as a line graph representing the time to fall from the rotarod counted as seconds in the trial and test (**D–F**). Data are presented as Mean ± SEM, and levels of significance are shown as *p* (*) values [*p < 0.05; *t-test*, Chi-squared test]

We performed an open-field test following different parameters to study the behavioral impact of blast injury and the influence of PUFA on general locomotor dysfunction associated with mTBI. We observed that Saline-Blast mice traveled a slightly shorter distance than the sham mice, and this distance did not change with PUFA feeding. Units of distance traveled are comprised of separate bouts of locomotion called progression segments. In both sham and blast mice, PUFA decreased the number of progression segments but increased their length and duration, particularly in blast mice (Fig. 4A). Of note, the 5 th and 95 th percentiles of progression duration were lower in Saline-Blast mice. (Fig. 4A). Additionally, Saline-Blast mice demonstrated slower movement compared to Saline-Sham mice in terms of mean speed (Fig. 4A). However, PUFA administration improved the speed of both PUFA-Sham and PUFA-Blast mice above that of Saline-Blast mice. Although speed increased in PUFA-fed mice, PUFA-Blast did not demonstrate more distance traveled than the Saline-Blast mice because PUFA-Blast mice spent more time stationary in what are known as lingering episodes. The diversity level, which is defined as the average distance between any two stops while considering the spatial and temporal distribution of stops and their durations, was significantly lower in Saline-Blast mice compared to their sham counterparts. In contrast, the diversity level of PUFA-Blast mice was higher than that of Saline-Blast mice (Fig. 4A). We also analyzed three additional open-field measures: center rest proportion, stops per distance, and path curvature. Mice typically avoid the center of an arena due to their instinct to seek cover. However, Saline-Blast mice spent more time in the center, suggesting reduced anxiety—likely due to impaired risk avoidance (Fig. 4A). PUFA-Blast mice spent significantly less time in the center than Saline-Blast mice.

Saline-Blast mice exhibited more frequent lingering episodes (stops per distance) than Saline-Sham mice; however, the duration of these lingering episodes was not significantly different (Fig. 4A). PUFA-Blast mice paused less often than Saline-Blast mice, but their pauses were significantly longer. This, in addition to fewer progression segments, accounts for why PUFA-Blast mice covered less distance. Furthermore, Saline-Blast mice had more difficulty walking in a straight line compared to sham mice, whereas PUFA-Blast mice exhibited improved linear movement (Fig. 4A).

### Tail Suspension Depression Evaluation

Depression due to mTBI was assessed via a tail suspension test after 1 month of blast injury. A longer duration of immobility during the tail suspension indicates higher levels of depression. Figure 4C illustrates the percentages of immobility over the 5 min of suspended time, with cumulative immobility measured at one-minute intervals. Significant differences were observed in the percentages of immobility between the Saline-Blast and PUFA-Blast mice. Saline-Blast mice exhibited a higher immobility score across the 5 min of the test, indicating elevated levels of depression compared to PUFA-Blast mice (Fig. 4B). No changes were observed for the sham mice, in either saline-fed or PUFA-fed groups (Fig. 4B). We then subtracted the values from the sham group from those of the blast group for both saline-fed and PUFA-fed mice. For saline-fed mice, the difference between sham and blast was significantly positive throughout the test duration, indicating that blast values were higher. In contrast, for the PUFA-fed mice, the Sham-Blast difference remained close to zero, indicating no significant difference in depression scores in PUFA-fed mice throughout the 5-min test (Fig. 4C).

### The Rotarod Motor Test

The rotarod motor test was conducted after one month of blast injury among four subsets of mice (Saline-Sham, Saline-Blast, PUFA-Sham, and PUFA-Blast), with 6–8 mice in each group (3–6 months old, including equal numbers of males and females). The mice underwent three training trials followed by three test trials, measuring the latency to fall (in seconds). As anticipated, TBI led to decreased rotarod performance in both Saline and PUFA-fed mice, indicating motor deficits (Fig. 4D). However, the PUFA-Blast mice displayed slight improvement by the third training trial and in the first two test trials (Fig. 4D). By the third test trial, all groups performed similarly. To evaluate the impact of PUFA supplementation regardless of TBI status, we combined the sham and blast groups separately for males and females (Fig. 4E and F). PUFA-fed males outperformed saline-fed males. Similarly, PUFA-fed females showed improved performance, with the most significant benefits observed during the test sessions (Fig. 4E and F). These results suggest that PUFA supplementation enhances motor recovery with benefits observed in both males and females.

### Assay for Oculomotor Nucleus

**Fig. 5:**
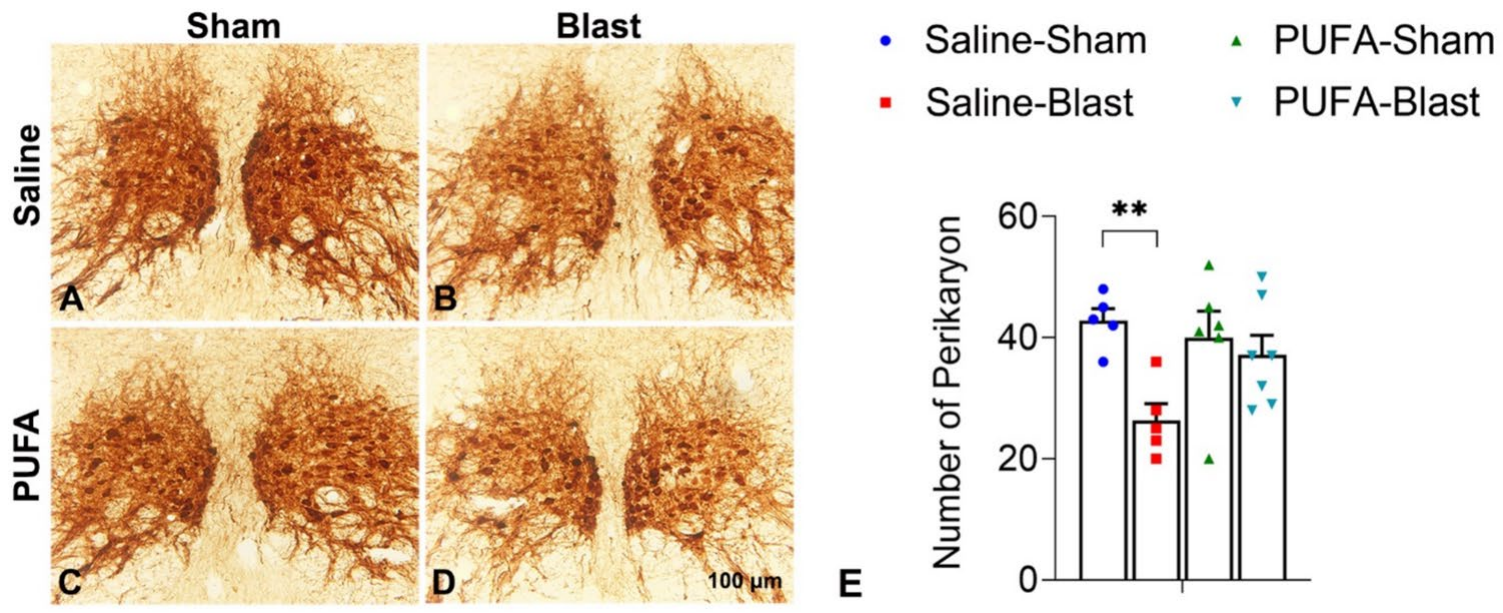
Immunohistochemical analysis of oculomotor neurons with choline acetyltransferase (ChAT) immunostaining at midbrain level in sections from saline-fed (Saline) and PUFA-fed (PUFA) mice with and without mild TBI. Representative images of ChAT-immunolabeling of the oculomotor nucleus of Saline-Sham, Saline-Blast, PUFA-Sham, and PUFA-Blast littermates (**A–D**). The bar diagram represents the number of perikarya at the oculomotor nucleus in the brain sections of Saline-Sham, Saline-Blast, PUFA-Sham, and PUFA-Blast mice (**E**). Perikarya are represented by the dark-stained nuclei, and neuronal loss was estimated by counting these nuclei. Data are presented as Mean ± SEM (*n* = 5–7), and levels of significance are shown as *P values* (*p < 0.05, *t-test*)

We performed an immunohistochemical analysis of the oculomotor nucleus, specifically focusing on the expression of choline acetyltransferase (ChAT) to understand the effect of TBI on the motoneurons. The images of ChAT immunostaining demonstrate various degrees of staining between the groups (Fig. 5A–D), with significant differences in the quantity of ChAT-positive perikarya (neuronal cell bodies) in the oculomotor nucleus. Specifically, the number of perikarya was reduced in the Saline-Blast mice compared to the Saline-Sham group, while the PUFA-fed mice exhibited similar numbers of perikarya between the sham and blast conditions (Fig. 5E). Overall, our findings indicate that mild TBI can reduce both the area and the quantity of motoneurons in the oculomotor nucleus and highlight the protective role of polyunsaturated fatty acids in preserving motoneuron integrity following TBI.

### Assays for Microglia Activity

**Fig. 6:**
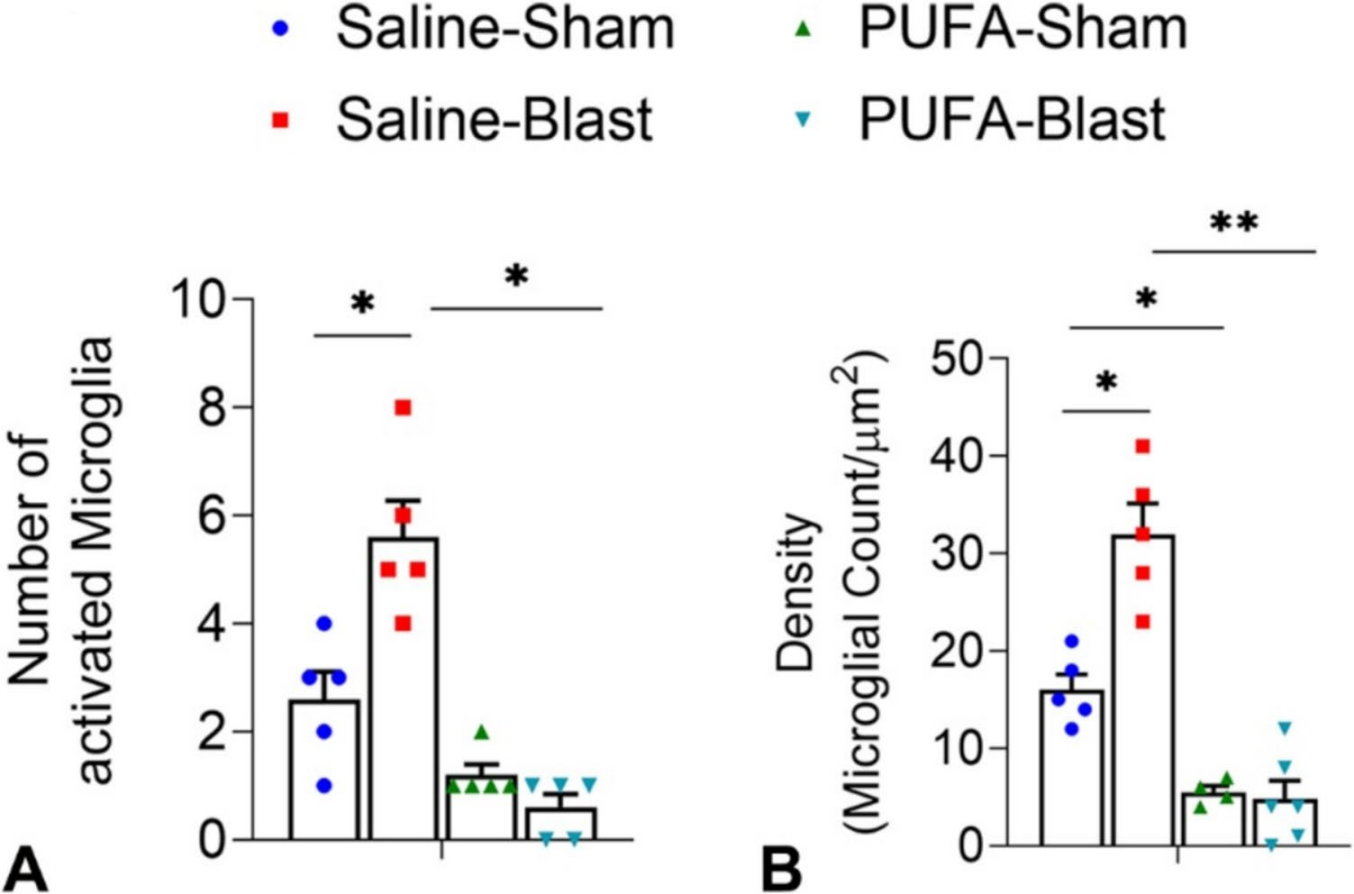
Analysis of microglial activation in the optic tract by immunostaining for IBA1 from saline-fed (Saline) and PUFA-fed (PUFA) mice with and without mild TBI. The histogram shows the number of active microglia (**A**) and density (microglia/µm.^2^) (**B**) at the right side of the optic tract in the brain section. Data are presented as Mean ± SEM, and levels of significance are shown as *P values* (*n* = 5–6 each for Saline-Sham, Saline-Blast, PUFA-Sham, PUFA-Blast; *p < *0.05*, **p < *0.01*, *t-test*)

We utilized immunohistochemical analysis using IBA1 protein as a marker to detect activated microglia. Our data indicate a significant increase in the number of activated microglia in the optic tract area of the Saline-Blast group compared to the Saline-Sham group (Fig. 6A), suggesting that blast injury induces microglial activation. These patterns were mirrored in the PUFA-fed mice, with a significant increase of microglia activity observed in the PUFA-Blast mice compared to the PUFA-Sham mice (Fig. 6B). However, PUFA-fed mice (both Sham and Blast) did not exhibit a marked activation of microglia (Fig. 6B). The lack of a marked increase in the PUFA groups further indicates the potential neuroprotective or anti-inflammatory effect of a PUFA after a blast injury. This finding could have therapeutic implications for conditions of neuroinflammation.

### Sphingomyelinase Assay

**Fig. 7:**
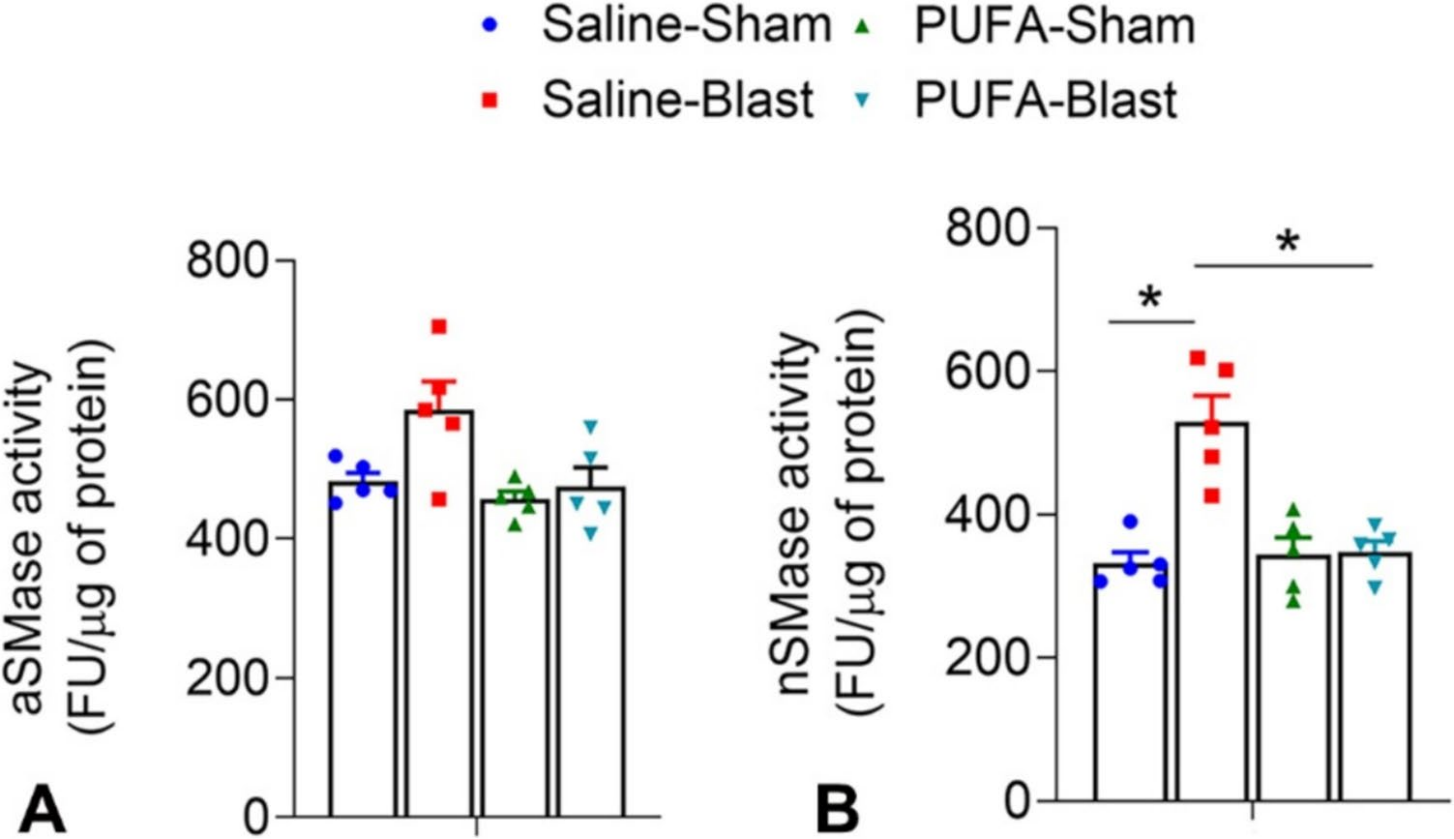
Enzyme activity of sphingomyelinase (SMase) (fluorescence unit/ug of protein) in the brain tissues of saline-fed (SF) and PUFA-fed (PUFA) mice treated with 0-psi (sham) or 50-psi (blast) one month after TBI. No significant changes were noted in acidic SMase (aSMase) activity across groups (**A**). A significant increase in neutral SMase (nSMase) activity was noticed in Saline-Blast mice compared to their sham counterpart (**B**). Data are presented as Mean ± SEM, and levels of significance are shownas *P values* (*n* = 5 each for Saline-Sham, Saline-Blast, PUFA-Sham, PUFA-Blast; *p < *0.05*, *t-test*)

Sphingomyelinases, both acidic and neutral Sphingomyelinase (aSMase and nSMase, respectively) assay were measured from the protein isolated from brain tissues after 35 days of post-TBI in all four conditions. We noticed no significant changes in aSMase activity across the different groups of mice (Fig. 7A). However, the data demonstrated a significant increase in nSMase activity in Saline-Blast mice compared to Saline-Sham mice (Fig. 7B), suggesting that TBI results in an elevated breakdown of sphingomyelin in the brain. PUFA-fed mice revealed resistance to the activation of nSMase activity after brain injury. These findings imply that a diet rich in PUFA may offer some protection against the biochemical changes associated with neuronal damage from TBIs.

### Oral n3-PUFA Can Modulate SPL Profile

**Fig. 8:**
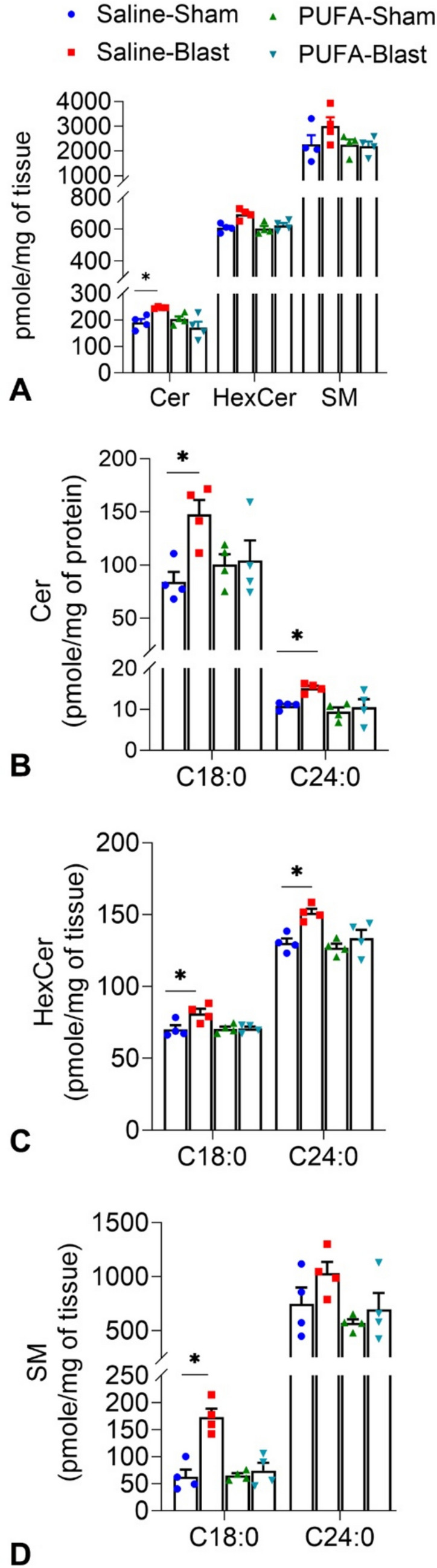
Analysis of Sphingolipid profile in the brain tissues of Saline-fed (Saline) and PUFA-fed (PUFA) mice treated with 0-psi (sham) or 50-psi (blast) 1 month after TBI. Histogram represents the level (pmol/mg of protein) of Ceramide (Cer), Hexosyl-Ceramide (HexCer), and Sphingomyelin (SM) in the brain tissues of Saline-Sham, Saline-Blast, PUFA-Sham, PUFA-Blast mice (**A**). An increased level of C18 and C24 Cer species was noticed in the brain tissues of Saline-Blast mice (**B**). An increased level of C18 HexCer species was noticed in the brain tissues of Saline-Blast mice (**C**). An increased level of C18 SM species was noticed in the brain tissues of Saline-Blast mice (**D**). Data are presented as Mean ± SEM and levels of significance are shown as *P values* (*n* = 4 Saline-Sham, 5 Saline-Blast, 4 PUFA-Sham, 4 PUFA-Blast; *p < *0.05*, *t-test*)

MS/MS analysis of SPL revealed that the total levels of ceramide were higher in Saline-Blast mice brains, with a significant increase in ceramide levels compared to Saline-Sham mice brains (Fig. 8A). This is consistent with increased nSMase activity from TBI (Fig. 7B). PUFA-fed brains did not show changes in these levels after TBI, further suggesting preventive effects of elevated EPA in the brain. Among the various individual species, we found specific increases in certain ceramides (C18:0 and C24:0), monehexosylceramides (HexCer, C18:0), and sphingomyelins (C18:0) in the brains of Saline-Blast mice (Fig. 8B–D). These increases were not observed in the brains of PUFA-fed mice after TBI. These findings support the conclusion that TBI leads to alterations in SPL metabolism and dietary PUFA-mediate increase of EPA in the brains might have therapeutic implications for treating mild TBI by counteracting these alterations.

## Discussion

TBIs can trigger a cascade of biochemical and cellular responses that lead to prolonged cognitive, sensory, and motor dysfunctions [7, 8]. In this study, we aimed to comprehensively examine both the biochemical (SPL and microglia activation) and functional pathological responses following mild TBI (vision, emotional regulation, and movement), particularly in the context of a dietary intervention involving n-3 PUFA. Limitations of this study include a small sample size and the administration of PUFA both before and after TBI, which may limit its direct applicability to post-injury treatment. Future studies will explore the efficacy of n-3 PUFA administered solely after TBI to better assess its therapeutic potential in a clinical setting. However, our findings strongly suggest that n-3 PUFA supplementation would remain beneficial even when initiated after TBI, offering protection against sensory, motor, and emotional impairments. Combined with our prior study’s results, it becomes evident that the administration of n-3 PUFA can be neuroprotective in TBI due to their ability to modulate SPL metabolism, reduce neuroinflammation, and preserve neuronal function [12].

We hypothesize that lipids can act as both pathological and protective agents in mTBI, particularly focusing on the interplay between n-3 polyunsaturated fatty acids (n-3 PUFA) and SPL metabolic pathways. Lipids are essential to regulating cellular function, from maintaining membrane architecture, cell signaling, and energy storage to cellular homeostasis. Given that the brain is a lipid-rich organ composed of high levels of glycerophospholipids, cholesterol, and SPL, any dysregulation in these components can contribute to neuroinflammation and neurodegenerative diseases. In recent years, SPLs have gained recognition as critical mediators in the neuronal injury response, with their altered metabolism linked to neuroinflammatory and neurodegenerative processes [18]. In particular, ceramides and sphingomyelins have been implicated in these neurodegenerative processes, triggering neuroinflammation, spreading neurotoxic proteins, and causing oxidative stress [40, 41]. Furthermore, polyunsaturated fatty acids play a crucial role in the brain’s neuroinflammatory, neurotrophic, and neuroprotective processes. For example, n-3 PUFA (EPA and DHA) act as neuroprotective agents, whereas n-6 PUFA (Arachidonic acid, 20:4n6) acts as a neuroinflammatory agent [42].

In the present experimental condition, significantly higher levels of ceramides were found in Saline-Blast mice brain tissue compared to Saline-Sham mice. However, PUFA-fed brain tissues did not exhibit changes in these levels post-TBI, indicating a potential protective effect (Fig. 8). The alteration in the SPL profile in EPA-enriched brains may be due to n-3 PUFA’s (especially EPA’s) impact on SPL metabolism, indicating a potential crosstalk between these two distinct types of lipid metabolic factors. This was further supported by our finding of reduction of neutral SMase activity in the brain tissues of PUFA-Blast mice (Fig. 7). Building on our previous study, these results suggest that dietary n-3 polyunsaturated fatty acids (PUFA) may represent an effective therapeutic strategy for managing inflammatory sphingolipid (SPL) levels. In the acute phase of brain injury in the experimental mouse model of mTBI, an increased level of short-chain Cer and elevated sphingomyelinase activity was noticed [43]. These findings indicate that changes in ceramide subspecies resulting from TBI could be useful as potential pathological biomarkers in the clinical management of TBI.

N-3 PUFAs have been associated with the modulation of neuroinflammatory processes by reducing inflammatory factors and the activation of microglia. Previous studies indicate that n-3 PUFA exert anti-neuroinflammatory effects by influencing microglial polarization, inhibiting key inflammatory signaling pathways, and simultaneously activating neuroprotective pathways [44]. A significant increase in the inflammatory factors, such as Il-1α (interleukin-1α), Il-1β (interleukin-1β), Tnf-α (tumor necrosis factor-α), Cox-2 (cyclooxygenase 2), and iNos (inducible nitrite oxide synthase), has been observed in the brain tissue of injured mice compared to those fed a PUFA-rich diet [45]. However, systemic elevation of n3-PUFA in transgenic mice demonstrates reduced pan-inflammatory factors, namely, Tnf-α, Il-6 (interleukin-6), iNos, Nlrp3 (NLR family pyrin domain containing3), Cxcl1 (C-X-C-motif ligand 1), Cxcl10 (C-X-C-motif ligand 10) [12]. Furthermore, studies have demonstrated that optic nerve axon loss following TBI is associated with microglial activation, and preventing this activation correlates with reduced optic nerve axon loss [10, 46]. In the present study, the marked reduction in microglial activation within the optic tract of PUFA-fed mice post-TBI (Fig. 6) underscores the potential of n-3 PUFA to suppress the inflammatory cascade that typically follows TBI.

Microglial activation can also contribute to the development of depression and cognitive defects through the production of proinflammatory mediators [12, 47]. In this study, we observed that PUFA-Blast mice exhibited lower levels of depression-like behavior compared to Saline-Blast mice in the tail suspension test. This difference may be attributed to an elevated endogenous level of EPA in the brain tissue (Fig. 4). Furthermore, clinical evidence supports the idea that elevated levels of EPA can have antidepressant effects. A clinical trial comparing ethyl-EPA to standard therapy demonstrated that EPA was more effective in treating depression, supporting the role of EPA in alleviating depression [48]. Together, these findings emphasize the potential of n-3 PUFA as a therapeutic target in reducing post-TBI-depression.

Lastly, our study also investigated the impact of increased n-3 PUFA on retinal and oculomotor nucleus degeneration, which are associated with major visual consequences in mTBI. TBI can significantly impair visual acuity and everyday visual-motor tasks due to the intimate link between the brain and the eyes, with effects that may be enduring [33]. TBIs can lead to distinct pathological changes in the eye, including photoreceptor degeneration [10, 49]. A previous study demonstrated that n-3 PUFA modulates retinal very-long-chain (VLC)-PUFA levels, which are essential for retinal function and structure. Dietary supplementation with n-3 PUFA has been shown to counteract the decrease in VLC PUFA associated with diabetic retinopathy, suggesting their potential role in preserving retinal integrity [50]. In our ERG study, animals fed with n-3 PUFA-enriched fish oil displayed minimal deviation in a-wave and b-wave amplitudes. On the other hand, saline-fed mice demonstrated significantly reduced levels of a- and b-wave amplitude after TBI (Fig. 3). This indicates that dietary supplements containing n-3 PUFA may help mitigate the impact of blast injuries on retinal dysfunction. This is particularly compelling when juxtaposed with previous observations of Mondal et al. [12] and Desai et al. [22]. They observed a significant difference in a-wave among those mice reared with an n3-PUFA-deficient diet compared to the n3-PUFA-adequate diet. These findings suggest that n-3 PUFA may safeguard photoreceptor function and, by extension, retinal integrity against oxidative stress and neuronal damage induced by TBI. Furthermore, we observed the protection from ChAT^+^-perikaryon loss in the brain section after blast injury in PUFA-fed mice compared to the saline-fed mice (Fig. 5). This is a crucial finding, as mild TBI commonly leads to oculomotor deficits, such as issues with accommodation, convergence, and saccadic eye movements [51]. The maintenance of ChAT-positive neurons in the oculomotor nucleus of transgenic-PUFA mice and PUFA-fed mice suggests that n-3 PUFA can play a vital role in preserving the integrity and function of these neurons, thereby contributing to better visual outcomes after TBI.

## Conclusion

Overall, this study provides strong evidence that dietary supplementation with n-3 polyunsaturated fatty acids can offer significant protective effects against the multi-faceted deficits induced by mild TBI. By integrating these findings with the existing literature, our research demonstrates that the therapeutic benefits of n-3 PUFA in TBI scenarios are mediated through the modulation of lipid metabolism, reduction of neuroinflammation, and preservation of neuronal and retinal function. Our study strongly aligns with our previous study results, indicating the role of n-3 PUFA in reducing the elevation of SPL metabolites. Future research should reveal the specific mechanisms underlying each of these effects and translate them into effective clinical applications for human TBI recovery protocols.

## Data Availability

No datasets were generated or analysed during the current study.

## Declarations

### Animals

Animal studies approved by the UTHSC IACUC committee. Approval # 19–0104.
